# Towards a Quantitative OCT Image Analysis

**DOI:** 10.1371/journal.pone.0100080

**Published:** 2014-06-13

**Authors:** Marina Garcia Garrido, Susanne C. Beck, Regine Mühlfriedel, Sylvie Julien, Ulrich Schraermeyer, Mathias W. Seeliger

**Affiliations:** 1 Division of Ocular Neurodegeneration, Institute for Ophthalmic Research, Centre for Ophthalmology, Tuebingen, Germany; 2 Section of Experimental Vitreoretinal Surgery, Centre for Ophthalmology, Tuebingen, Germany; Justus-Liebig-University Giessen, Germany

## Abstract

**Background:**

Optical coherence tomography (OCT) is an invaluable diagnostic tool for the detection and follow-up of retinal pathology in patients and experimental disease models. However, as morphological structures and layering in health as well as their alterations in disease are complex, segmentation procedures have not yet reached a satisfactory level of performance. Therefore, raw images and qualitative data are commonly used in clinical and scientific reports. Here, we assess the value of OCT reflectivity profiles as a basis for a quantitative characterization of the retinal status in a cross-species comparative study.

**Methods:**

Spectral-Domain Optical Coherence Tomography (OCT), confocal Scanning-La­ser Ophthalmoscopy (SLO), and Fluorescein Angiography (FA) were performed in mice (*Mus musculus*), gerbils (*Gerbillus perpadillus*), and cynomolgus monkeys (*Macaca fascicularis*) using the Heidelberg Engineering Spectralis system, and additional SLOs and FAs were obtained with the HRA I (same manufacturer). Reflectivity profiles were extracted from 8-bit greyscale OCT images using the ImageJ software package (http://rsb.info.nih.gov/ij/).

**Results:**

Reflectivity profiles obtained from OCT scans of all three animal species correlated well with ex vivo histomorphometric data. Each of the retinal layers showed a typical pattern that varied in relative size and degree of reflectivity across species. In general, plexiform layers showed a higher level of reflectivity than nuclear layers. A comparison of reflectivity profiles from specialized retinal regions (e.g. visual streak in gerbils, fovea in non-human primates) with respective regions of human retina revealed multiple similarities. In a model of Retinitis Pigmentosa (RP), the value of reflectivity profiles for the follow-up of therapeutic interventions was demonstrated.

**Conclusions:**

OCT reflectivity profiles provide a detailed, quantitative description of retinal layers and structures including specialized retinal regions. Our results highlight the potential of this approach in the long-term follow-up of therapeutic strategies.

## Introduction

Vision starts in the retina located at the posterior part of the eye. “Rete”, the Latin origin of its name standing for “net”, connotes with two important properties, a two-dimensional layer structure and a multitude of connections. Indeed, the retina is composed of several heavily interconnected neuronal layers, each with a specific functional property from the light reception to signal processing and data reduction [1].

In contrast to the rather similar principal organization of the retina in layers, its topography varies substantially between mammalian species, presumably due to evolutionary influences of the environmental conditions [2], [3]. In humans and non-human primates (NHPs), a central region of high visual acuity, the macula, has evolved, whereas most other species have a more or less clearly expressed visual streak that is usually located at the separation of the upper and the lower retina. This configuration is believed to follow the basic visual needs of each species, namely high-acuity vision of the horizon, low-sensitivity vision of the (bright) sky, and high-sensitivity vision of the (relatively dim) ground.

Traditionally, fundus photography and angiography have been used to assess macroscopic retinal structure and its changes in disease, whereas fine details were merely accessible via *ex vivo* methods like histology and immunohistochemistry. It was a major breakthrough in ophthalmic diagnostics when Optical Coherence Tomography (OCT) was first introduced as a novel tool for *in vivo* visualization of retinal layers [4]–[6]. The resolution of third generation models of OCT equipment that became available a few years later finally turned out to be sufficient for use in rodent models of retinal disease [7], [8]. A particular asset for experimental research is the option to follow the course of disease and/or monitor the effects of a therapeutic intervention over time in individual eyes [7], [9].

Technically, OCT provides cross-sectional images based on the reflective properties of the investigated sample [4], [5]. A single measurement of the reflectivity versus depth at one specific location is called A-scan, whereas the composition of an image by alignment of several consecutive A-scans is called B-scan [10].

A typical B-scan shows several, often alternating bands of low and high reflectivity, as plexiform layers have a higher level of reflectivity than nuclear layers [11]. However, these bands and the retinal layers associated with them vary in their extent with the topographical position in the retina, and this is additionally species-dependent as mentioned above. So far, automated segmentation procedures have still not reached a satisfactory level of performance, which is why in the majority of cases simply a qualitative evaluation is performed.

In this work, we use the layer reflectivity in OCT images as a function of scan depth (similar to A-scan data) for a quantitative analysis of the retina of three different species.

Experimental quantifications based on A-scans have been performed in the past, but have not led to a widespread use of respective approaches [12]–[15]. Nevertheless, we show here that the information contained in A-scan data is very helpful for the robust quantification of changes in health and disease, and that respective parameters have the potential for excellent quantitative biomarkers bypassing the need for an accurate segmentation of the B-scan images.

## Materials and Methods

### Ethics Statement

All procedures in rodents were performed according to the German laws governing the use of experimental animals and were previously approved by the local authorities (Regierungspraesidium Tuebingen), which are in accordance with the ARVO statement for the Use of Animals in Ophthalmic and Visual Research.

The OCT data from Monkeys was taken from a dataset recorded as part of a separate study at Covance Laboratories (Muenster, Germany).

### Animals

The present study includes three animal models, mice, Gerbils, and Cynomolgus monkeys. In the rodent part, four individual animals per line or species were used (pigmented C57BL/6 wild type mice, non-pigmented BALB/c mice, and gerbils (*Gerbillus perpallidus*)). Rodents were kept under a 12 h∶12 h light-dark cycle (60 lux) and they had free access to food and water. Mice were anesthetized with ketamine (66,7 mg/kg) and xylazine (11,7 mg/kg) and their pupils were dilated with tropicamide eyedrops (Mydriaticum Stulln; Pharma Stulln, Stulln, Germany) before image acquisition. Gerbils were anesthetized following the indications of a previous study with this specie carried out by our group [16].

The OCT data of five Cynomolgus monkeys (*Macaca fascicularis*, ages 10 to 15 years, supplied by Nafovanny, Vietnam) were recorded as part of a separate study [17], and used here to construct the reflectivity profiles. Primates were anesthetized with ketamine hydrochloride (10 mg/kg, Ketavet; Pharmacia GmbH, Erlangen, Germany) plus xylazine (2 mg/kg, WDT, Garbsen, Germany). Their pupils were dilated (tropicamide, phenylephrine, Novartis, Siemens, Germany) and their corneae anesthetized (oxybuprocainhydrochloride, Novartis).

### Scanning-Laser Ophthalmoscopy (SLO)

Retinal structures of the anesthetized animals were visualized via SLO imaging with a HRA 1 and HRA 2 (Heidelberg Engineering, Heidelberg, Germany) according to previously described procedures [18]. Briefly, HRA 1 and HRA 2 systems feature lasers in the short (visible) wavelength range (488 nm in both and 514 nm in HRA 1 only), and also in the long (infrared) wavelength range (795/830 nm and 785/815 nm). The 488 and 795 nm lasers are used for fluorescein (FLA) and indocyanine green (ICG) angiography, respectively.

### Spectral Domain Optical Coherence Tomography (SD-OCT)

SD-OCT imaging was performed in the same session as cSLO and it was carried out with a Spectralis HRA+OCT (Heidelberg Engineering GmbH, Heidelberg, Germany). This device features a superluminescent diode at 870 nm as low coherence light source. Scans are acquired at a speed of 40.000 scans per second and each two-dimensional B-scan contains up to 1536 A-scans. [7]. The images were taken with the equipment set of 30° field of view and with the software Heidelberg Eye Explorer (HEYEX version 5.3.3.0, Heidelberg, Germany).

### Image Acquisition and Image Analysis

In order to define a reproducible reflectivity profile in mouse and gerbil, the position of the retinal fundus image was standardized. To achieve that, the position of the eye was adapted until the optic disc was exactly in the center of the fundus region visualized with the SLO module of the Spectralis, and all OCT scans were acquired in this position. In analogy, the visual streak in gerbils and the fovea in cynomolgus monkeys was positioned in the center of the retinal image. In cynomolgus monkeys, additional reflectivity profiles were extracted from OCT scans taken from a retinal region rich in nerve fibres [19], close to the optic disc.

OCT scans were exported and converted with Corel Draw X3 (Corel Corporation, Ottawa, ON Canada) into a 8-bit greyscale images. Images were then processed with the ImageJ software package (http://rsb.info.nih.gov/ij/), and reflectivity profiles were extracted from ten adjacent parallel lines which crossed perpendicularly the OCT scan from the upper layer, the ganglion cell layer to the bottom layer, the retinal pigmented epithelium. For a visual representation of the underlying statistics of the data, reference lines indicating the 5, 50 and 95 quantiles were generated.

### *Ex vivo* Analysis of Retinal Morphology

Mice and gerbils were sacrificed upon completion of experiments and their eyes were marked and enucleated for histological analysis. They were fixed in 2,5% glutaraldehyde prepared in 0.1 M cacodylate buffer and processed as previously described [20]. Subsequently, semi-thin sections (0,5 mm) were obtained and counterstained with methylene blue and were posteriorly analyzed using a light microscope (Axiovision, Zeiss, Jena, Germany).

## Results

### Generation of OCT Reflectivity Profiles

Mammalian retinae differ somewhat in their morphological landmarks. To understand commonalities and differences in respective OCT data, a comprehensive in vivo examination using scanning-laser ophthalmoscopy (SLO), fluorescein angiography (FLA), and optical coherence tomography (OCT) was done in three mammalian laboratory species, mice, gerbils and cynomolgus monkeys. OCT reflectivity profiles were then generated by averaging ten adjacent pixel columns from the greyscale image data with the ImageJ software package as described in methods. The SLO examination included the native red-fee (RF; 513 nm), infrared (IR; 830 nm), and autofluorescence (AF; 488 nm) modes. Retinal vasculature was assessed after injection of fluorescein dye via 488 nm wavelength laser with a barrier filter at 500 nm. The results are summarized in the following sections.

### OCT Reflectivity Profiles in Mice

**Figure 1:**
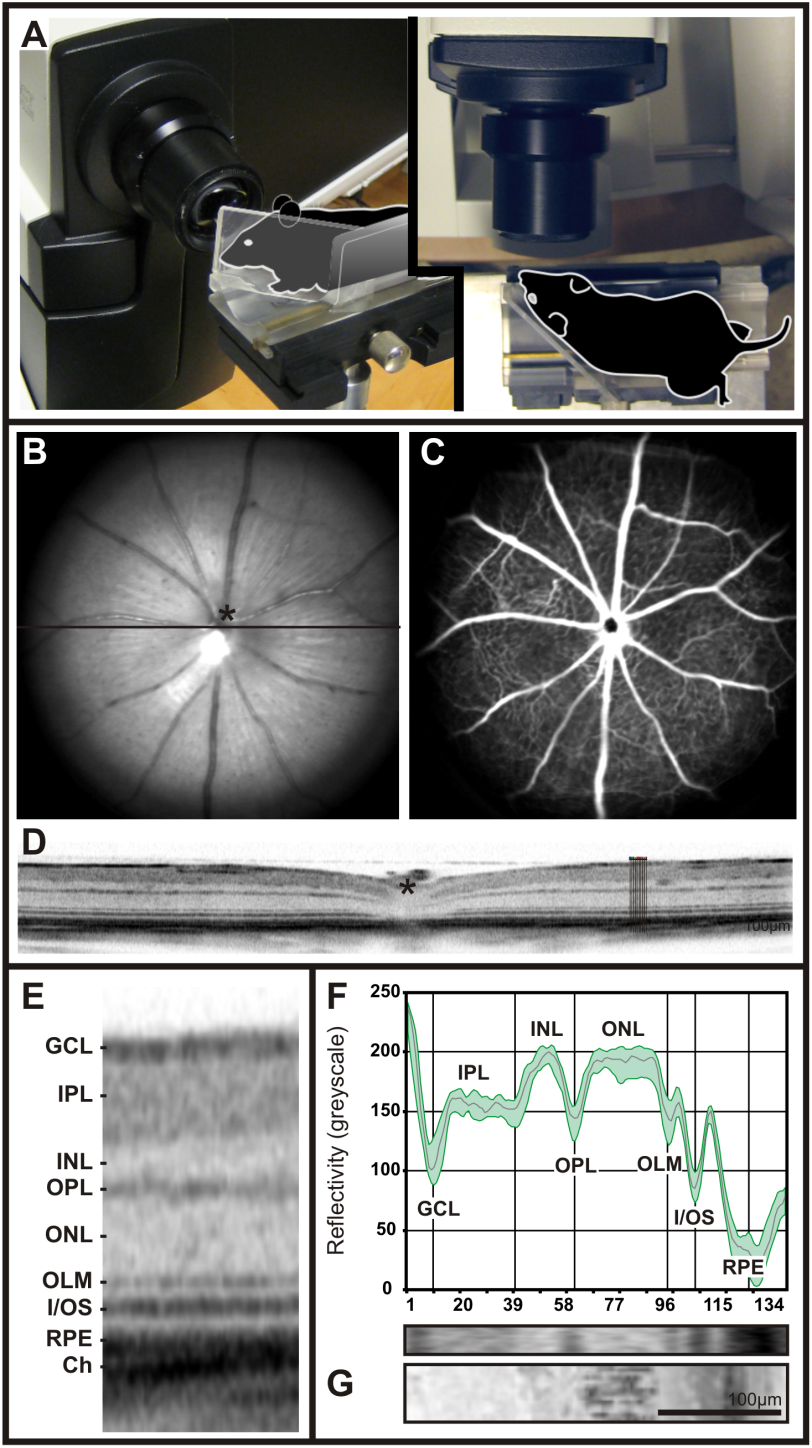
Set-up to visualize the mouse retina: retinal morphology, layer composition and OCT reflectivity profile (A–F). Illustrative representation of the mouse placed in front of the Spectralis camera (**A**). Mouse fundus native image at 513 nm (**B**) and retinal angiography image following fluorescein dye injection using a barrier filter at 488 nm (**C**). Retinal layer composition by means of an OCT horizontal scan through the optic disc (asterisk) and schematic depiction of the 10 longitudinal adjacent pixel lines from which reflectivity profiles were extracted (**D**). Blow up of a section from the OCT scan indicating the retinal layers (**E**). Corresponding OCT reflectivity profiles from “D” and assignation to the different OCT bands as well as correlation with histology (**G**). **Abbreviations:** GCL, ganglion cell layer; IPL, inner plexiform layer; INL, inner nuclear layer; OPL, outer plexiform layer; ONL, outer nuclear layer; I/OS, inner/outer segment border; RPE, retinal pigmented epithelium.

**Figure 2:**
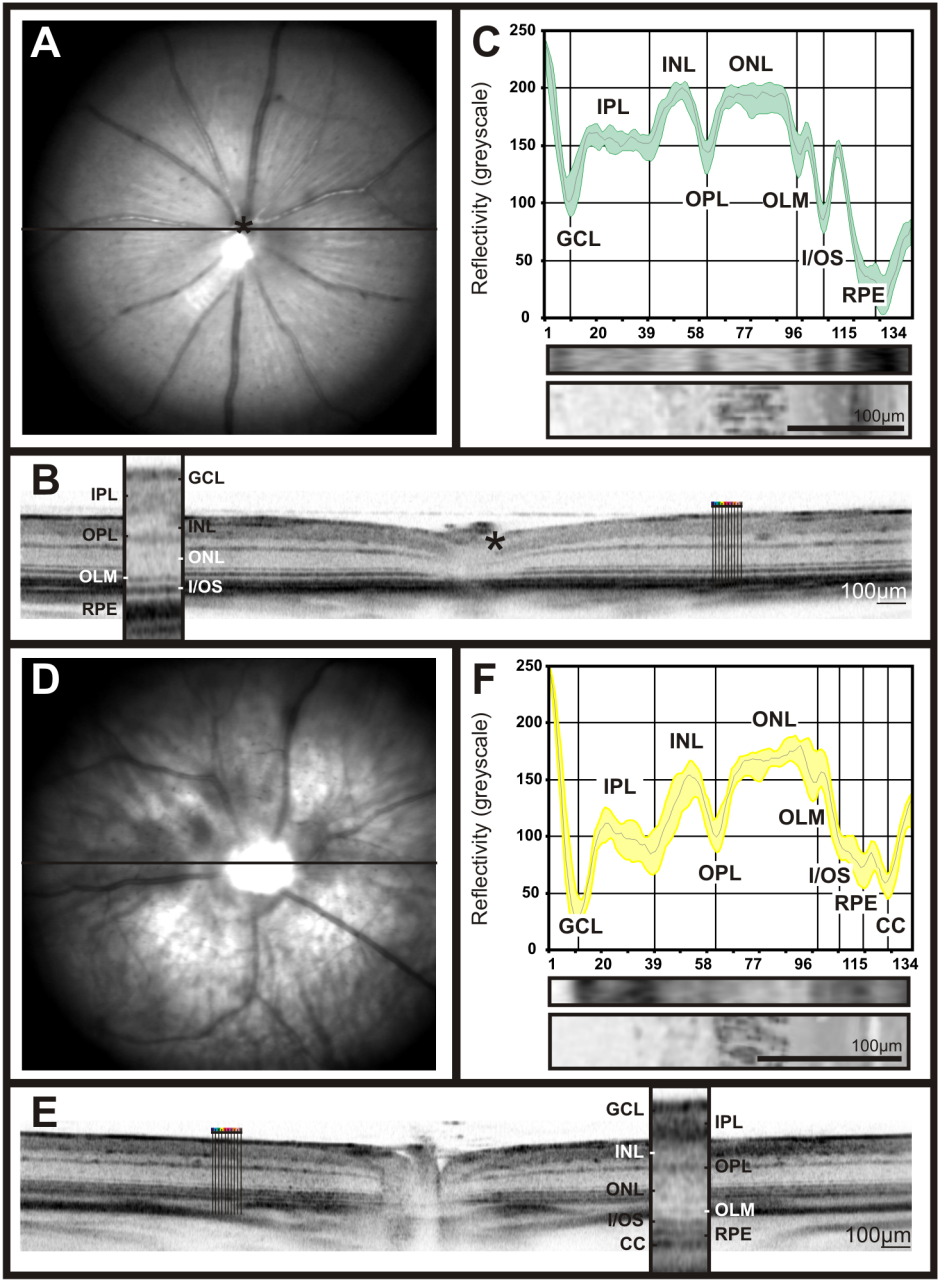
Retinal imaging and OCT reflectivity profiles of two control lines. Fundus native imaging at 514(**A, D**), horizontal OCT scan (**B, E**) and OCT reflectivity profiles were acquired from C57BL/6 pigmented (**A–C**) and BALB/c non pigmented mice (**D–F**).

Retinal image data using scanning-laser ophthalmoscopy (SLO), fluorescein angiography (FA), and optical coherence tomography (OCT) were obtained from pigmented C57Bl/6 and non-pigmented BALB/c mice. Typically, low greyscale values were found in regions corresponding to the nerve fibre layer (NFL), inner plexiform layer (IPL), outer plexiform layer (OPL), outer limiting membrane (OLM), the border between inner segment and outer segment (I/OS), and the retinal pigmented epithelium (RPE). In contrast, high greyscale values were typical for the inner nuclear layer (INL) and the outer nuclear layer (ONL). In this study, nuclear layer greyscale values in both mouse strains averaged about 175±15 units, whereas plexiform layers ranged around 150±15 units (Fig. 1F; Fig. 2 C, F).

Although the murine retina appears less topographically structured than that of many other mammals, there are still several structural differences concerning e.g. cell number and distribution, chemical gradients, and visual pigment distribution [3]. To assess whether or not such topographical differences affect OCT reflectivity properties, representative profiles were extracted from OCT scans taken from the dorsal, ventral, nasal and temporal parts of C57BL/6 mouse retinas. However, no substantial differences were found (Fig. S1).

Another important factor in retinal imaging is the degree of pigmentation, as it determines the amount of absorbed light in a wavelength-dependent manner. In this work, we addressed the influence of the degree of pigmentation via a comparison of data from heavily pigmented C57Bl/6 mice, featuring a relatively high melanin content in RPE and choroid, with those from non-pigmented BALB/c mice. As already known from SLO en face imaging work, melanin-rich structures reduce the transmission of light in a wavelength-dependent fashion [18], and although the infrared range is least affected by that, there are still perceivable effects in OCT [8]. The comparison between the two models revealed a generally increased scan depth when pigmentation was low, together with a better differentiation of bands in the outer retina/RPE region (Fig. 2E). These bands are presumably associated with retinal epithelium, choriocapillaris, choroid, and sclera (Fig. 2F).

### OCT Reflectivity Profiles in Gerbils

**Figure 3:**
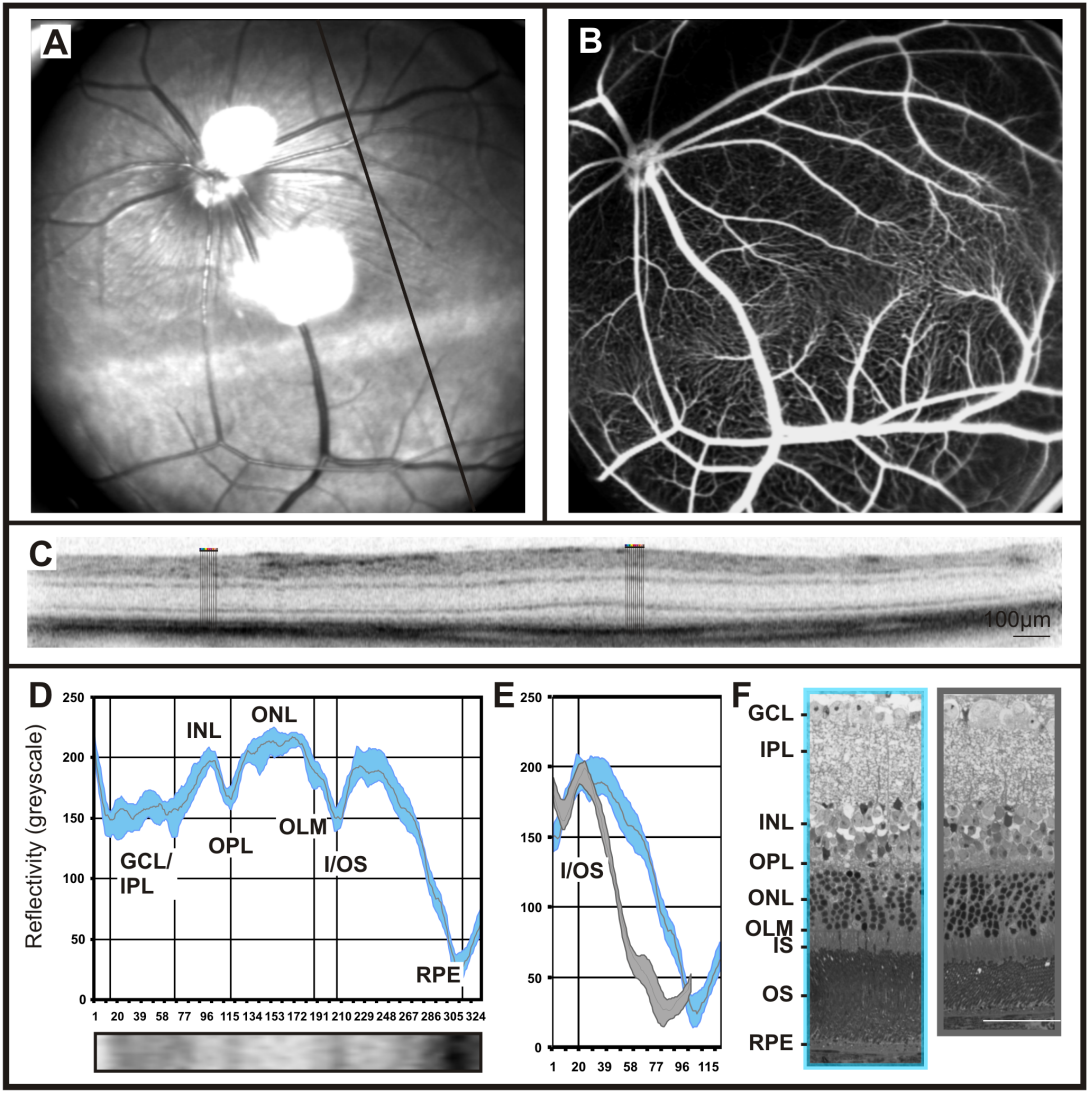
Retinal imaging and OCT reflectivity profile in gerbils. Native fundus image at 514(**A**). Using fluorescein angiography a characteristic ramification of the capillary net along the visual streak can be observed (**B**). OCT scan through the visual streak reveals changes in the layering in comparison to the rest of the retina (**C**). OCT reflectivity profile from 10 longitudinal adjacent lines at the level of the visual streak and correlation to the retinal layers (**D**). Overlay of the OCT reflectivity profiles extracted from the visual streak (blue) versus the non visual streak regions (grey) (**E**). Histological work-up showing structural differences between the visual streak (blue) and other retinal areas (grey) (**F**).

Gerbils are rodents that have a much more topographically structured retina than mice or rats, which has been attributed to their different circadian activity. Retinal image data using scanning-laser ophthalmoscopy (SLO), fluorescein angiography (FA), and optical coherence tomography (OCT) were obtained the same way as in mice. In this study we found that the principal layering structure and the reflectivity profiles were similar to that of most other rodent species, i.e. plexiform layers form zones of higher reflectivity, and nuclear layers bands of lower reflectivity (Fig. 3 D, E). However, there were strong topographical differences in that Gerbils have a strongly expressed visual streak (VS). The VS is, according to current knowledge, an area of increased visual performance for objects on the horizon (Fig. 3A), and thus both morphologically and functionally resembles some features of the human macula. In particular, this includes a characteristic vascular pattern (Fig. 3B), as well as an increased thickness of the photoreceptor layer. Since a similar, but less strongly expressed retinal pattern was found in the unstriped soudanian grass rat (*Arvicanthis ansorgei*) [21], one may speculate that this is common to day active rodents in general.

The recorded OCT reflectivity profiles enabled the quantification and allocation of the corresponding topographical differences in retinal layers, particularly with respect to the visual streak in the dorsal part of the retina (Fig. 3D, E). In this region, the number of photoreceptors is increased, as was confirmed histologically (Fig. 3F). The increased number of photoreceptors is reflected in the OCT reflectivity profile by a much broader extent of the outer segment band when compared to more peripheral regions.

### OCT Reflectivity Profiles in Non-human Primates (NHPs)

**Figure 4:**
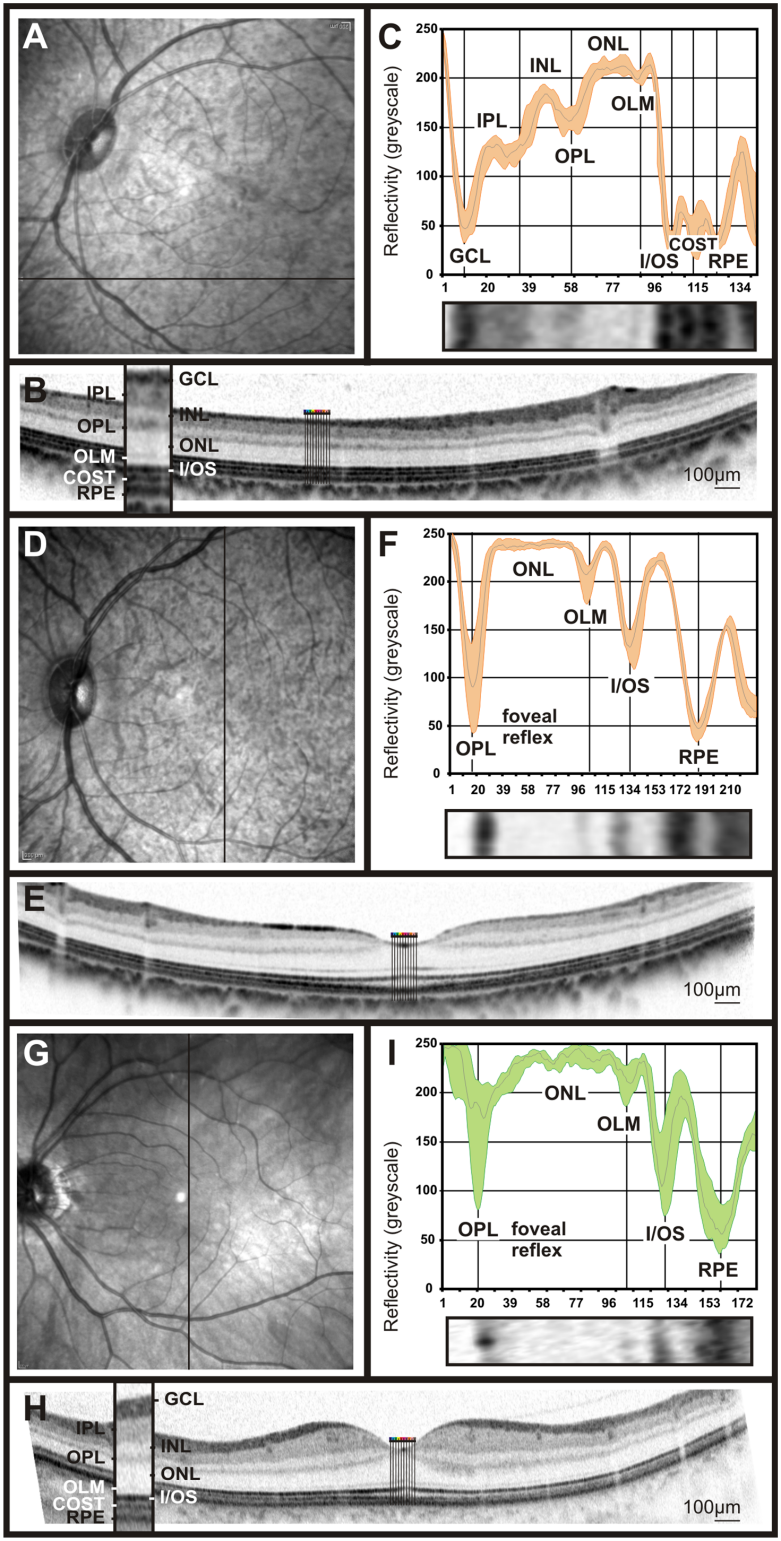
Retinal imaging and OCT reflectivity profile in cynomolgus monkeys and comparison to humans. SLO native fundus imaging in non-human primates (**A**, **D**) and one of the researcher’s eye (**G**). The solid bar indicates the origin of the OCT scan. A representative OCT scan taken from the ventral retina (**B**), the fovea of the non-human primate (**F**) and the researcher’s eye, (**H**). OCT reflectivity profiles from B, F and H were extracted and assigned to the retinal layers of cynomolgus monkeys fovea and extrafoveal regions (**F** and **C**, respectively) and the human fovea (**I**).

**Figure 5:**
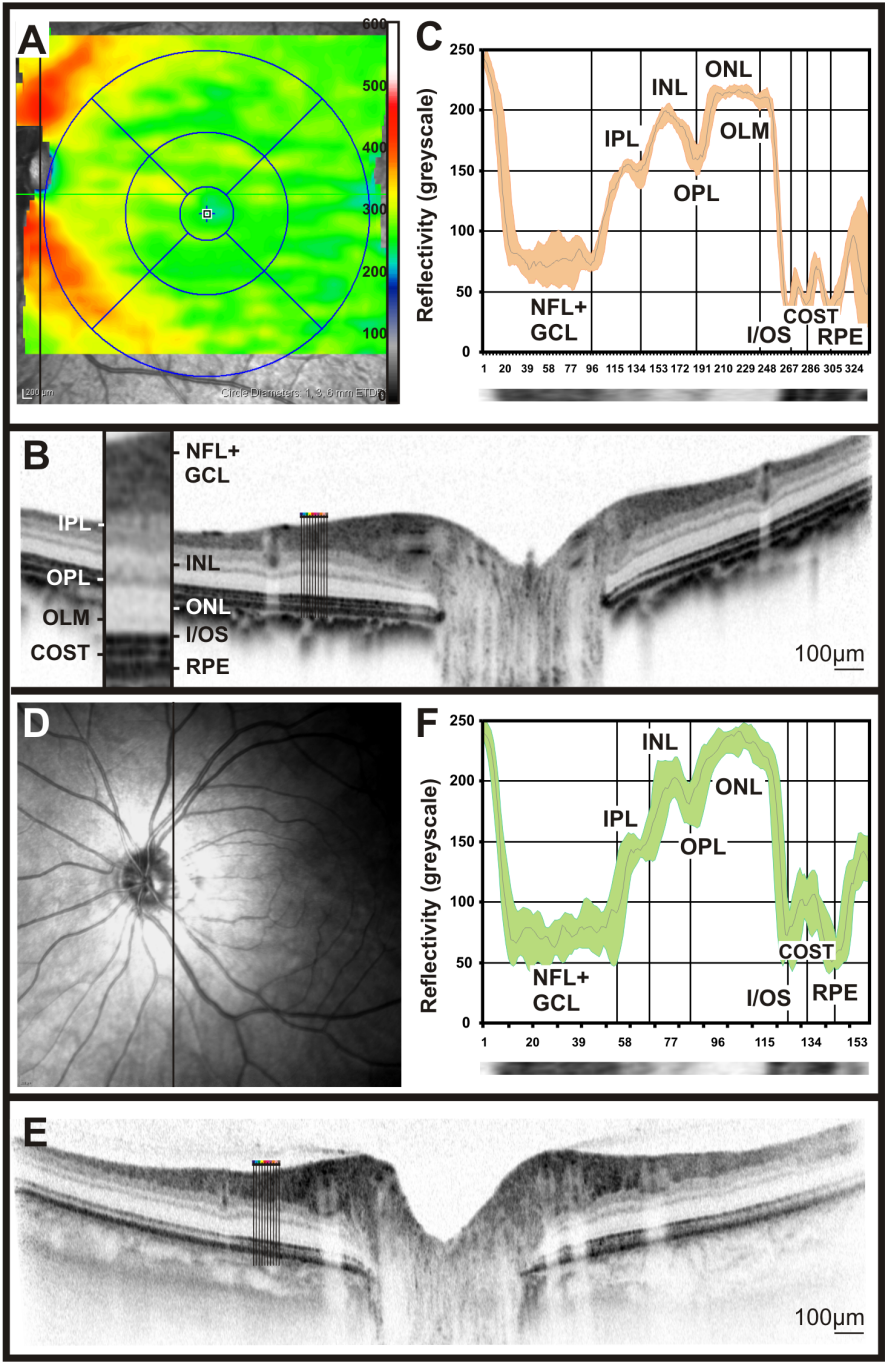
Comparison between the NFL appearance in cynomolgus monkeys and humans by means of OCT reflectivity profiles. Retinal thickness profile map of a cynomolgus monkey indicates where the thickest areas of the retina are located to, presumably due to the abundance of nerve fibers (**A**). SLO imaging in one of the researcher’s eye (**D**). Retinal layering via OCT imaging was performed across the central retina in cynomolgus monkeys (**B**) and compared to that of humans (**E**). OCT reflectivity profiles from a region rich in nerve fibers were acquired from cynomolgus monkeys (**C**) and that of human (**F**).

Non-human primates, due to the presence of a fovea, have an even more topographically structured retina. Again, retinal image data using scanning-laser ophthalmoscopy (SLO), fluorescein angiography (FA), and optical coherence tomography (OCT) were obtained to assess their retinal characteristics. Interestingly, reflectivity profiles from cynomolgus monkeys allowed for a further differentiation of the outer retina, as in extrafoveal regions an additional peak in the reflectivity profile, between the I/OS and the RPE peaks, was detected (Fig. 4C and 5D, G). This peak is thought to belong to the cone outer segment tips (COST) [22], [23]. Further, the reflectivity profiles were in accordance with the anatomical changes in the macular area, leading to a reduction and eventually the absence of the bands corresponding to GCL, IPL, and INL. However, the OPL, ONL, I/OS and RPE layers were clearly visible (Fig. 4D–F). When exactly centered on the fovea, the reflectivity curve started with a strong peak representing the foveal reflex (Fig. 4F, [24]). In general, the observed reflectivity profile showed a remarkable resemblance to human data (Fig. 4G–H). Nevertheless, some differences were detected, e.g. the ONL peak was wider in human subjects than in NHPs (Fig. 4I).

The retinal nerve fibre layer (NFL) is mainly formed by ganglion cell axons en route to the optic disc [19]. We gave special attention to the reflectivity pattern of this layer, since alterations in the number of nerve fibres are the basis for several optic dystrophies. Glaucoma is one of the most important diseases in this group, and although there are yet no sufficiently established criteria for detection and monitoring of this disorder, the assessment of NFL thinning is currently a common clinical test [25]. First, we compared the NFL appearance in cynomolgus monkey to that in human subjects (Fig. 5A–C vs. D–F). As part of this comparison, we generated a thickness map from volume scans with 97 B-Scans at 30 µm intervals centred to the fovea, which revealed an increase on the retinal thickness mainly at the sites where major blood vessels are located (Fig. 5A). However, the NFL quantification based vertical OCT scans around the optic disc, together with the reflectivity profiles extracted from them, suggested that a major portion of this thickness is true fibre increase and only a minor portion may be attributed to vessels directly (Fig. 5B & C, E & F). In summary, the comparison of OCT reflectivity profiles between NHPs and human subjects did not reveal major differences, so that this technique is well suited for the follow-up of NHPs in preclinical trials.

### Use of Reflectivity Profiles in the Evaluation of Therapeutic Interventions

**Figure 6:**
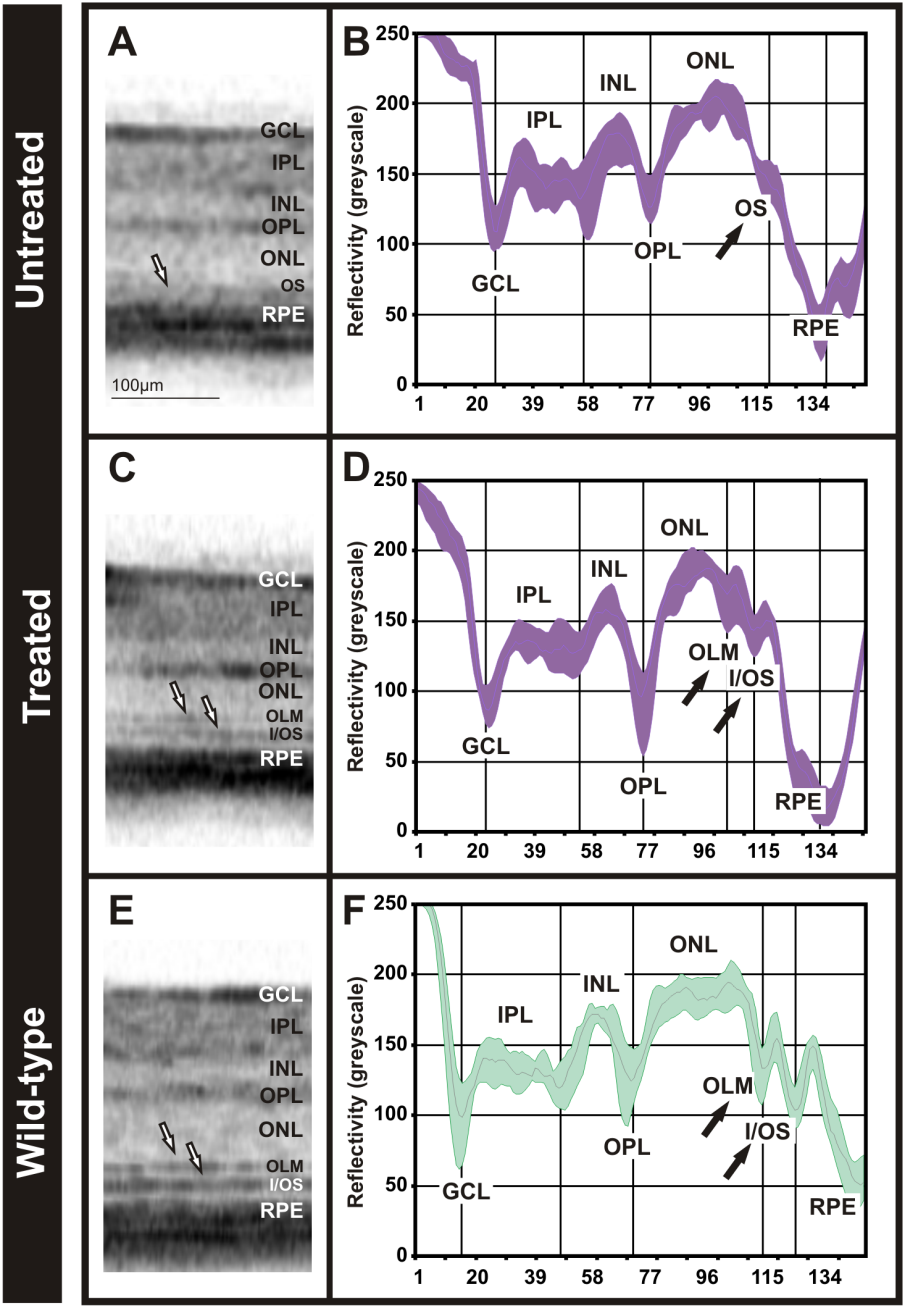
Comparison of retina layer composition and OCT reflectivity profile after a gene therapy approach in the Cngb1 *knock-out* mouse. Representative OCT sections of the untreated eye (**A**), treated (**C**) in the Cngb1 *knock-out* mouse and in a C57BL6 control mouse (**E**). OCT reflectivity profiles from the untreated eye (**B**), treated (**D**) and control (**F**).

The progress in molecular therapy of retinal diseases has led to a number of therapeutic approaches, implemented so far mainly in animal disease models and only partly in human clinical trials. In diseases that are accompanied by an alteration or degeneration of tissues, quantification of OCT data may be a valuable biomarker for the post-treatment follow-up. Here, we assessed this follow-up in a model of Retinitis Pigmentosa (RP) lacking the gene encoding the rod nucleotide-gated channel subunit CNGB1 [26]. Like in RP, a knock-out of CNGB1 leads to a mildly progressive degeneration of rods and subsequently of cones [27]. In early stages, rod cells are preserved but do not develop regular outer segments. Recently, we were able to restore morphology and function in the Cngb1 knock-out mouse by means of adeno-associated virus (AAV)-mediated gene therapy [28]. The OCT imaging follow-up of interventions applied in early disease stages (sixty days after injection) revealed that in the treated eye, the injected region (in our approach roughly one third of the retina) showed signs of a substantial rescue, presenting as a persistence of photoreceptor outer segments and maintenance of a regular retinal layering, whereas in the untreated eye no regular outer segment-related layers were found (Fig. 6C vs. A). In OCT reflectivity profiles from those respective areas, substantial differences were found (Fig. 6D vs. B). In the untreated eye, the two peaks corresponding to the OLM and the I/OS border, usually found at approximately 175±9 and 150±7 greyscale units, were absent; merely a minor OLM band remained visible (Fig. 6B, D). However, the regular peaks were present in the treated eye. Our results corroborate the restoration of the rod CNG channel [28], and show the potential for this diagnostic tool as a biomarker in the follow-up of therapeutic trials in retinal diseases.

## Discussion

In this work, we assess the potential of OCT reflectivity profiles as a basis for a quantitative characterization of the retinal morphology in vivo. Since we feel this approach may have a general value in the interpretation and quantification of mammalian OCT data, we chose a cross-species comparative study design.

Our results show that such quantitative OCT analyses are very well suited to capture and numeralize similarities and differences in the retina of three laboratory species with a different degree of topographic structuring (Fig. 1–4). Our work closely relates to human studies [12], [13], [15], [29].

The characteristic pattern of the mammalian reflectivity profiles may also help to better fine-tune automated segmentation algorithms. It is a long-standing problem that, because retinal anatomy and layering in health as well as in disease is complex and variable, automated segmentation procedures have not yet reached a satisfactory level of performance.

As a first step in this study, we generated a basic reflectivity profile of standard mouse lines, the C57BL/6 line representing pigmented strains, and the BALB/c line representing non-pigmented ones. Each part of the profile was matched to a corresponding retinal structure in ex vivo morphology (Fig. 1F and 2F). As presumed, we found that variations in the retinal pigment content do influence the detection of the underlying anatomical structures and their representation in the reflectivity profile (Fig. 2F). By acquiring several reflectivity profiles from different fundus locations, we could demonstrate that the comparatively minor topographical differences across the mouse retina (e.g. cell distribution, opsin gradient) did not substantially manifest in OCT reflectivity profiles (Fig. S1).

In a next step, we recorded a standard reflectivity profile of another rodent model, the gerbil (Fig. 3D, E). Gerbils are primarily diurnal (activity during the day and sleeping at night). Their body size lies between that of mice and rats, but their retinal organisation is very different from those primarily nocturnal species. Most obvious in this regard is the well-expressed visual streak, a specialized retinal region that resembles many features of the human macula [16]. The visual streak is represented in the native fundus image as a high reflective band located to the dorsal part of the retina (Fig. 3A). Based on the reflectivity profile, we were able to show that the visual streak region is characterized by an elongation of the crest corresponding to the photoreceptor outer segments (Fig. 3D, E). Another typical landmark in gerbils is the characteristic pattern of the retinal vasculature (Fig. 3B, further details in [16]). The relatively high similarity of the vascular organization with the human macula may render this class of rodents suitable models for experimental therapies in diseases with a strong vascular component like Diabetes Mellitus.

It is believed that the topographical differences in retinal morphology between different animal species have developed due to evolutionary pressure in their natural habitat. The driving force may be an advantage in the acquirement and processing of specific, vital visual information. Typical patterns associated with such an adaptation may include a difference in the number and type of retinal cells (e.g. photoreceptors, bipolar, or ganglion cells), the distribution and spectral sensitivity of visual pigments, or even variations in the vascular pattern [3]. It is believed that preferential day- or night-activity constitutes a major determina­tive factor in this context, and whether the animal’s role is rather prey or predator. Indeed, other day-active species like the unstriped soudanian grass rat (*Arvicanthis ansorgei*) are known to possess a retina rich in cones and a special organisation of those in the ONL [21], very well in agreement with our findings. More details regarding to the visual streak organi­sation in a predator like the cat with a so-called *area centralis* may be found elsewhere [30].

Finally, we turned to even further specialised retinas as found in primates and humans. These retinae are characterized by the presence of a *fovea* conferring high-acuity central vision. Based on Cynomolgus data, we produced a generic OCT reflectivity profile in non-human primates. All layers in foveal and non-foveal regions were successfully matched with the human counterparts (Fig. 4F and 4I). Also, the foveal reflex, featuring a total reflection, was detected in both primates and humans (Fig. 4F, I). The quantification of the retinal nerve fibre layer (RNFL) via reflectivity profiles pointed out a lower density of nerve fibres in cynomolgus monkeys In comparison to human retina (Fig. 5C, F). In diseases where the thickness of the NFL is altered (e.g. glaucoma), the assessment of the RNFL via reflectivity profiles may be a valuable biomarker in the follow-up of the disease.

In conclusion, we present here a way to use OCT reflectivity profiles for a quantitative description of retinal layers and structures including specialized retinal regions. The current developments of molecular therapies, particularly in retinal degenerations, will undoubtedly lead to a large number of clinical trials in the near future. In these imminent studies, the in vivo quantification of therapeutic effects over time will almost certainly include OCT data. In this work, we show that our technique is well applicable in both patients and experimental models. Our approach may in this situation constitute a valuable biomarker for the follow-up of therapeutic interventions in individual eyes.

## Supporting Information

Figure S1 **OCT reflectivity profiles from a C57BL/6 wild-type mouse.** Graphic representation of the layer reflectivity as a function of the scan depth extracted from representative OCT scans acquired from the dorsal, ventral, nasal and temporal parts of the retina and subsequent correspondence to the retinal layering.
(TIF)
