# Supplementary figures and images for: Towards a Quantitative OCT Image Analysis
Source: PLoS One. 2014 Jun 13;9(6):e100080. doi: 10.1371/journal.pone.0100080 (PMC4057353; doi:10.1371/journal.pone.0100080)

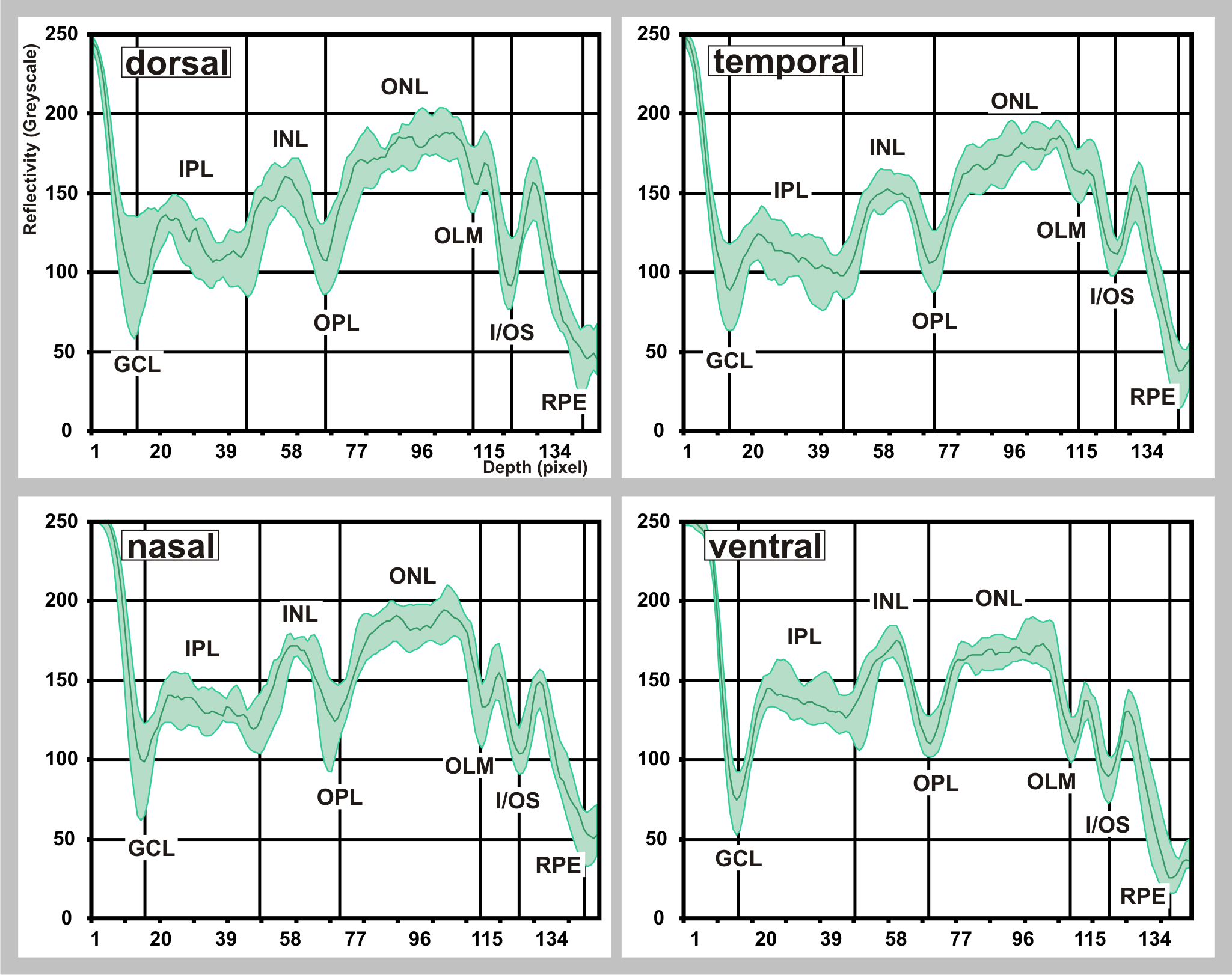

Supplement: Figure S1 — OCT reflectivity profiles from a C57BL/6 wild-type mouse. Graphic representation of the layer reflectivity as a function of the scan depth extracted from representative OCT scans acquired from the dorsal, ventral, nasal and temporal parts of the retina and subsequent correspondence to the retinal layering. (TIF) [file pone.0100080.s001.tif]
